# The complete mitochondrial genome of *Exopalaemon annandalei*

**DOI:** 10.1080/23802359.2018.1521307

**Published:** 2018-10-08

**Authors:** YiMing Yuan, YanLong He, ShouHai Liu, Xiao Ji, YuTao Qin, XiaoBo Wang

**Affiliations:** a East China Sea Environmental Monitoring Center of State Oceanic Administration, Shanghai, People’s Republic of China;; b Key Laboratory of Integrated Monitoring and Applied Technology for Marine Harmful Algal Blooms, SOA, Shanghai, People’s Republic of China

**Keywords:** Mitochondrial genome, *Exopalaemon annandalei*, Phylogenetic analysis

## Abstract

In this study, the complete mitochondrial genome (mitogenome) of *Exopalaemon annandalei* was amplified and analyzed. The mitogenome is 15,718 bp in length, encoding 13 protein-coding genes (PCGs), 22 tRNA genes, 2 rRNA genes, and a control region (CR). The nucleotide frequency of the mitogenome is as follows: A, 34.81%; C, 23.24%; G, 12.68%; and T, 29.25%. Seven kinds of the initiation codon and five kinds of termination codon are employed in the 13 PCGs. Phylogenetic analysis show *E. annandalei* to be in sister-relationship with *E. carinicauda*. The complete mitogenome sequence information of *E. annandalei* would play an important part in further studies on molecular systematics and phylogeny.

*Exopalaemon annandalei*, a common small-scale economic shrimp, is a unique species that is mainly distributed along coastal areas in China (Zhuang et al. 2008), with the characteristic of rapid propagation (Wu 1993). In view of the importance of *E. annandalei*, we sequenced its mitochondrial genome and used this to clarify its phylogenetic position.

In this study, the samples of *E. annandalei* (accession number: B0012-1) were collected from the East China Sea (121°59′10″E, 31°04′48″N). The specimens now were stored in East China Sea Environmental Monitoring Center. Partial sequences of *COI* and *16S rRNA* genes were amplified by PCR using universal primers. Based on these two PCR products, we designed two specific primer pairs to amplify two DNA fragments of the mitogenome of *E. annandalei*, respectively. Subsequently, these two PCR products were sequenced using Shot Gun Sequencing. Then all the sequences obtained were analyzed and assembled.

The complete mitogenome of *E. annandalei* (GenBank number MG787410) is a closed circular molecule, 15,718 bp in length. The gene content is similar to other animal mitochondrial genomes (Zhang et al. 2017), including 22 tRNA genes, 13 PCGs (COX1-3, NAD1-6, and 4L, COb, ATP6 and ATP8), two mitochondrial ribosomal RNAs (12S and 16S rRNA), and a control region (CR). Nucleotide frequency of the mitogenome is as follows: A, 34.81%; C, 23.24%; G, 12.68%; and T, 29.25%. The base composition has A + T bias, with the value of 67.07%. The ND1, ND4, ND4L, ND5, 16S rRNA, 12S rRNA, tRNA-Cys, tRNA-His, tRNA-Gln, tRNA -Leu, tRNA-Phe, tRNA-Pro, tRNA-Tyr, and tRNA-Val genes are encoded on the reverse strand of the mitogenome, whereas the remaining genes are encoded on the forward strand. Many different kinds of initiation codons exist in the 13 PCGs: six PCGs (ATP6, COX2, COX3, ND1, ND4L, and Cob) start with the ATG typical initiation codon, ND2 and ND5 genes share the ATA initiation codon, and the remaining five genes use ACG, ATC, ATT, GTG, and TTG as initiation codons. Both complete and incomplete stop codons are used in the protein-coding genes: COX1, COX2, and COb genes have incomplete stop codons T–/TA-, while the remaining genes have the stop codon TAA/TAG/TAC. All 22 tRNAs were identified in the *E. annandalei* mitogenome and range from 61 bp to 70 bp in length. The CR (934 bp) of the *E. annandalei* mitogenome is located between 12S rRNA and tRNA-Ile. We found microsatellite-like repeat (AT) _5_ and (TA) _5_ elements in the CR.

To clarify the phylogenetic relationship among decapoda, a phylogenetic tree was constructed using the Neighbor-Joining method (Figure 1) (Kumar et al. 2016). *E. annandalei* is grouped with *E. carinicauda* as sister species and located in the basal branch. *Palaemon gravieri* (Kim et al. 2017) and *Palaemon serenus* (Gan et al. 2016) have a close relationship with *E. annandalei*. This study would provide basic data for research on genetic analyses and evolutionary status of these species in the future.

**Figure 1.**
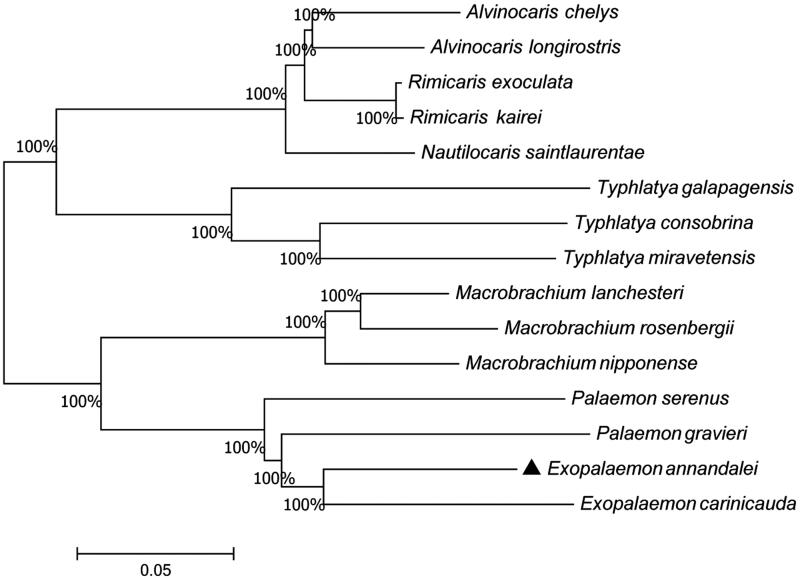
Neighbor-Joining tree of the amino acid sequences of all protein-coding genes in shrimps. The numbers on the nodes show the bootstrap percentages.
